# Analysis of transcriptomics data from COVID-19 patients: a pilot research

**DOI:** 10.1007/s12223-024-01130-x

**Published:** 2024-01-19

**Authors:** Dominik Hadzega, Klaudia Babisova, Michaela Hyblova, Nikola Janostiakova, Peter Sabaka, Pavol Janega, Gabriel Minarik

**Affiliations:** 1 Medirex Group Academy, Nitra, Slovakia; 2 Comenius University in Bratislava, Medical Faculty, Institute of Medical Biology, Genetics and Clinical Genetics, Špitálska 24, Bratislava, Slovakia; 3 Department of Infectology and Geographical Medicine, Faculty of Medicine, Comenius University in Brati-Slava, Bratislava, Slovakia

**Keywords:** RNA-seq, COVID-19, SARS-CoV-2, Gene enrichment analysis, Enriched pathways, Differentially expressed genes, Transcriptomics

## Abstract

During SARS-CoV-2 infection, the virus transforms the infected host cell into factories that produce new viral particles. As infection progresses, the infected cells undergo numerous changes in various pathways. One of these changes is the occurrence of a cytokine storm, which leads to severe symptoms. In this study, we examined the transcriptomic changes caused by COVID-19 by analyzing RNA-seq data obtained from COVID-19-positive patients as well as COVID-19-negative donors. RNA-seq data were collected for the purpose of identification of potential biomarkers associated with a different course of the disease. We analyzed the first datasets, consisting of 96 samples to validate our methods. The objective of this publication is to report the pilot results. To explore potential biomarkers related to disease severity, we conducted a differential expression analysis of human transcriptome, focusing on COVID-19 positivity and symptom severity. Given the large number of potential biomarkers we identified, we further performed pathway enrichment analysis with terms from Kyoto Encyclopedia of Genes and Genomics (KEGG) to obtain a more profound understanding of altered pathways. Our results indicate that pathways related to immune processes, response to infection, and multiple signaling pathways were affected. These findings align with several previous studies that also reported the influence of SARS-CoV-2 infection on these pathways.

**Supplementary Information:**

The online version contains supplementary material available at 10.1007/s12223-024-01130-x.

## Introduction

SARS-CoV-2 virus first emerged in December 2019 when it infected a patient hospitalized with the disease now known as COVID-19 (Wu et al. 2020). It subsequently spread worldwide causing a global pandemic (Cucinotta and Vanelli 2020). For this pandemic event, much more powerful tools were available to study and manage the outbreak than ever before. Next-generation sequencing capabilities helped scientists study the genetic code of the virus and its evolution over time (Oude Munnink et al. 2021).

One of these capabilities certainly represents the possibility to study how the human transcriptome in the tissue of an infected patient changes or how it differs in case of more severe symptoms. After infecting human tissue, SARS-CoV-2 alters normal host cell metabolism and signaling to adjust the intracellular environment for itself and its own replication. In general, this involves interfering with signaling pathways that regulate processes of DNA repair and replication, immune response, transcription, metabolism, cell cycle, and apoptosis. In particular, the pathways that are known to be altered include phosphoinositide 3-kinase (PI3K)/protein kinase B (AKT), Type I and III interferons, transforming growth factor-β (TGF-β), Toll-like receptors (TLR), and nuclear factor kappa light chain enhancer (NF-κB) pathways. Also, the altering of the Ca2 + signaling is believed to be part of the infection (Jamison et al. 2022). More severe phenotypes have been linked to excessive and uncontrolled immune response known as cytokine storms. It leads to the release of a large amount of pro-inflammatory cytokines into the blood-stream, followed by widespread inflammation and damage to tissues and organs (Henderson et al. 2020; Mehta et al. 2020; Wang et al. 2020).

This study is a pilot to our ongoing research on the impact of SARS-CoV-2 infection on the human transcriptome and bacterial transcript abundance (yet to be published) analyzed from RNA-sequencing of nasopharyngeal swabs. This study investigates the transcriptome profiles in COVID-19-positive and COVID-19-negative samples. We focused on differential gene expression and identification of pathways affected by the infection. Moreover, we compared patients infected with different SARS-CoV-2 variants.

## Materials and methods

### Study approval

Sample collection was performed as part of the clinical study approved by the Ethical Committee of Bratislava Self-Governing Region under the identifier 03228/2021/HF is-sued January 12, 2021. All patients completed questionnaires with relevant information regarding their health condition in relation to COVID-19 and signed informed consent.

### Samples

Nasopharyngeal swabs from patients suspected of having COVID-19 were obtained in two primary regimens. Firstly, patients with severe disease symptoms hospitalized at the partnering hospitals were enrolled in the study. Secondly, patients with mild or no disease symptoms were recruited in mobile testing facilities for SARS-CoV-2 by a company providing routine laboratory diagnostic services for the general population during the COVID-19 pandemic. Subgroups 1 to 4 were formed based on positivity and negativity of SARS-CoV-2 testing and the severity of COVID-19 symptoms according to the following scheme: group 1, positive with severe symptoms; group 2, positive with mild symptoms; group 3, no symptoms; group 4, negative. To classify cases, the WHO definition of symptoms was used (living guidance for clinical management of COVID-19, 2021 by World Health Organization). In total, 96 samples were included in the study (72 COVID-19-positive patients and 24 healthy sample donors). Nasopharyngeal swabs were collected from March 2021 to October 2022.

### Nucleic acid extraction

Nasopharyngeal swab specimens were collected from COVID-19 patients and individuals from control group and stored in the viRNAtrap collection medium (GeneSpector, Czech Republic) at 4 °C. Total RNA was extracted using Sera-Xtracta Virus/Pathogen Kit (Cytiva, UK) in line with the manufacturer instructions. Four hundred microliters of the nasopharyngeal swab medium was used for the extraction with a final elution volume of 50 μl. RNA was quantified with the Qubit™ RNA High Sensitivity Assay Kit (Invitrogen). RNA isolates were stored at − 80 °C.

### RT-qPCR

The presence of SARS-CoV-2 was determined by RT-qPCR using the COVID-19 Real-Time Multiplex RT-PCR Kit (Labsystems Diagnostics, Finland) and the ABI QuantStudio 6 RT-qPCR platform and Real-Time PCR System (Thermo Fisher, USA) utilizing the original manufacturers’ protocols. Amplification cycles threshold of Ct value < 40 was needed to evaluate the sample as positive.

### RNA library preparation and sequencing

The metatranscriptomic libraries were prepared using KAPA RNA HyperPrep Kit with RiboErase (HMR) (Kapa Biosystems, South Africa) in line with the original protocol of the manufacturer. For quantity and quality control of prepared libraries, we used a Qubit 1X dsDNA High Sensitivity Assay Kit on Qubit 3.0 (Invitrogen) and Agilent High Sensitivity DNA Kit on Agilent 2100 Bioanalyzer (Agilent) instruments. Sequencing of pooled libraries was performed on NextSeq 500 and NextSeq 2000 (Illumina) platforms using 2 × 75 or 2 × 100 paired-end sequencing setup, respectively.

### Quality control and data preparation for analysis

First step of any analysis of RNA-seq data is quality control and this step was done by FastQC v0.11.9(Andrews 2010). Reads were processed by Trimmomatic v0.39 (CROP:96 HEAD-CROP:10 LEADING:22 TRAILING:22 SLIDINGWINDOW:4:22 MINLEN:25 and our own set of adapter sequences were used in ILLUMINACLIP step) (Bolger et al. 2014). Parameters were chosen according to FastQC results.

### Reads mapping

After final affirmation of sufficient quality of reads by FastQC, reads were mapped to the human genome hg38 using the BWA-MEM algorithm v0.7.17 (Li 2013). Reads were mapped as paired set; otherwise, mapping parameters were set to default. The same procedure was applied on the SARS-CoV-2 genome. Mapping statistics were produced from a “.bam” outputs by Samtools flagstat (https://github.com/bahlolab/bioinfotools/blob/master/SAMtools/flagstat.md. (Accessed 23.05. 2023) n.d.).

### SARS-CoV-2 variant identification

For SARS-CoV-2 variant identification, we used the Galaxy pipeline, specifically “Mutation calling, viral genome reconstruction and lineage/clade assignment from SARS-CoV-2 sequencing data” (Maier and Batut 2023). For input, we used reads mapping on SARS-CoV-2 genome.

### Differentially expressed genes analysis

In search of human genes affected by the SARS-CoV-2 infection or the particular category of infection, we further analyzed the.bam file with human-mapped reads. Gene expressions were quantified by FeatureCounts v.2.0.1 (Liao et al. 2014). Statistical comparison was performed by the R instance of Deseq2 v.1.38.3. (Love et al. 2014). Genes in the condition of adjusted *p*-value < 0.1 were considered as significant hits. This is a default value according to DESeq2 manual (DESeq2 Manual (Accessed 23.05. 2023) n.d.).

### Identification of altered pathways

To find out which pathways were altered from the set of differentially expressed genes, we used the R instance of gProfiler2 v.0.2.1 (Kolberg et al. 2020). Genes found under *p*-value threshold 0.1 were used as a query for the analysis, ranked according to *p*-values and separated to down-regulated and up-regulated set. Databases of KEGG pathways, Wiki pathways, and Reactome were used in order to improve visibility; only the KEGG terms were selected for visualization. Set of genes which were part of differentially expressed gene analysis were set as custom background. Domain scope was set to “known.” For the visualization, barplot function in R was used.

## Results

In this study, we analyzed RNA-seq data from nasopharyngeal swabs. 96 samples were included in the study (72 COVID-19-positive patients and 24 negative donors). More detailed patient data are shown in Table 1.

**Table 1:** Patient details. Here, we show specific numbers of patients in the respective subgroups as well as their sex and age statistics

**Samples**	**Phenotype**	**Number of samples**		**Male/female**		**Age median**
**Negative**	-	24		12/12		33.5
**Positive**	Asymptomatic	24	72	14/10	40/32	42
	Mild	25		14/11		46
	Severe	23		12/11		60
**Positive with variants**	Alpha	24	36	14/10		46
	Delta	8		4/4		48.5
	Omicron	4		0/4		43

### SARS-CoV-2 sequences in our samples

We assigned SARS-CoV-2 in our samples with specific clade and the WHO variant name. Twenty-four samples were assigned as Alpha variant (clade 20I), 8 samples as Delta (clade 21 J), 4 samples as Omicron (clade 21L), one sample was assigned to the 20C clade, and one was reported as recombinant.

### Human transcriptome changes in COVID-19

**Fig. 1:**
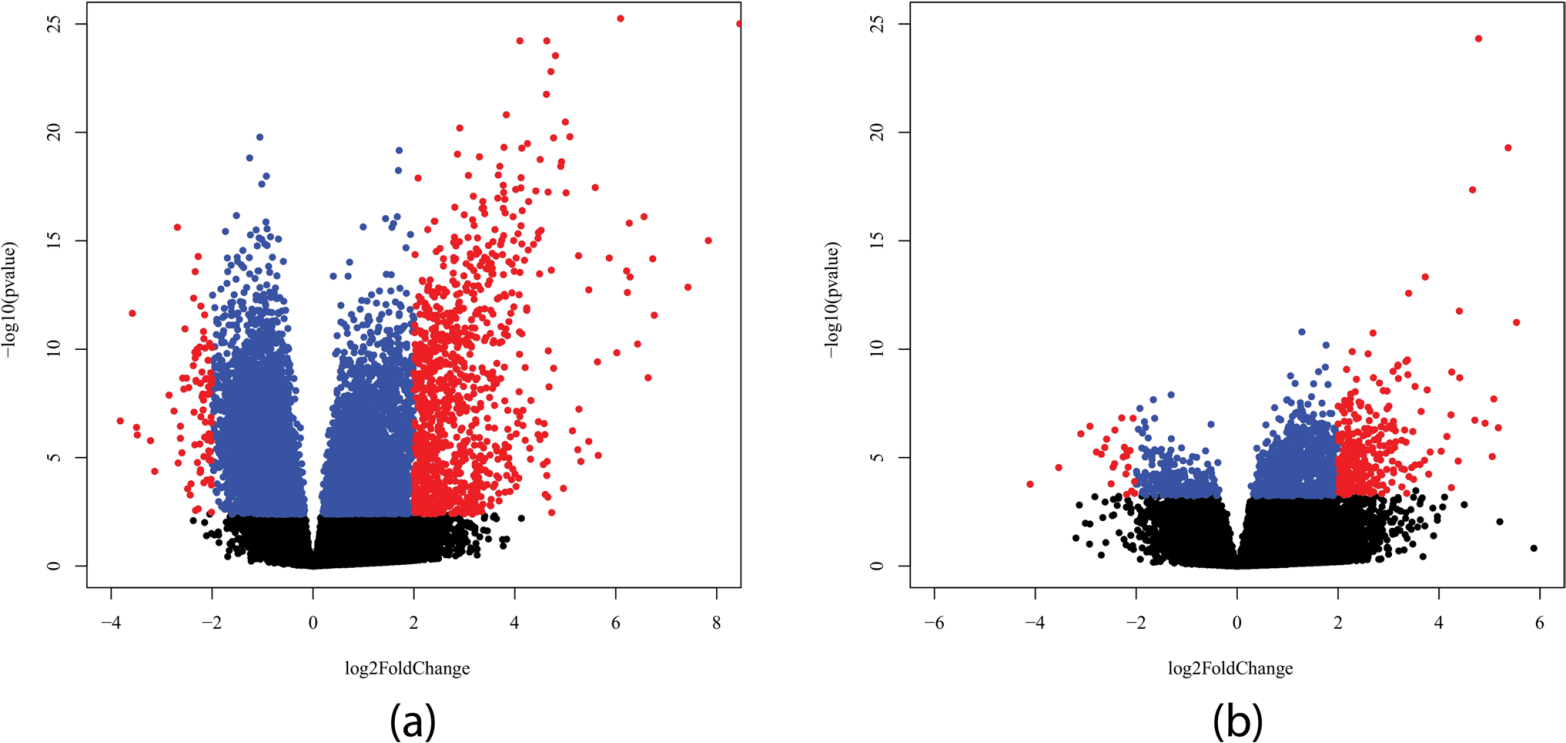
Volcano plots showing differential expression by plotting every gene as a dot positioned by log2 of its fold change (axis *x*) and − log 10 of its adjusted *p*-value (axis *y*). The colors show whether or not the gene passes through threshold requirements—blue (adj. *p*-value < 0.01) and red (adj. *p*-value < 0.01 and log2 fold change > 2). **a** Volcano plot visualizing differential expression—down- and up-regulated genes in the samples from COVID-19 patients. **b** Volcano plot visualizing differential expression between the samples from patients with severe symptoms (up-regulated right) compared to those with mild symptoms

According to our results, there were significant changes in the human transcriptome in COVID-19 patients. DESeq2 statistical test reported 16,365 genes with an adjusted *p*-value < 0.1 compared to COVID-19 negative controls (Supplementary Table 1). Four thousand five hundred thirty-nine genes were reported with a *p*-value < 0.1 when comparing mild and severe disease (Supplementary Table 2). Expression change profiles are shown as volcano plots in Fig. 1.

**Fig. 2:**
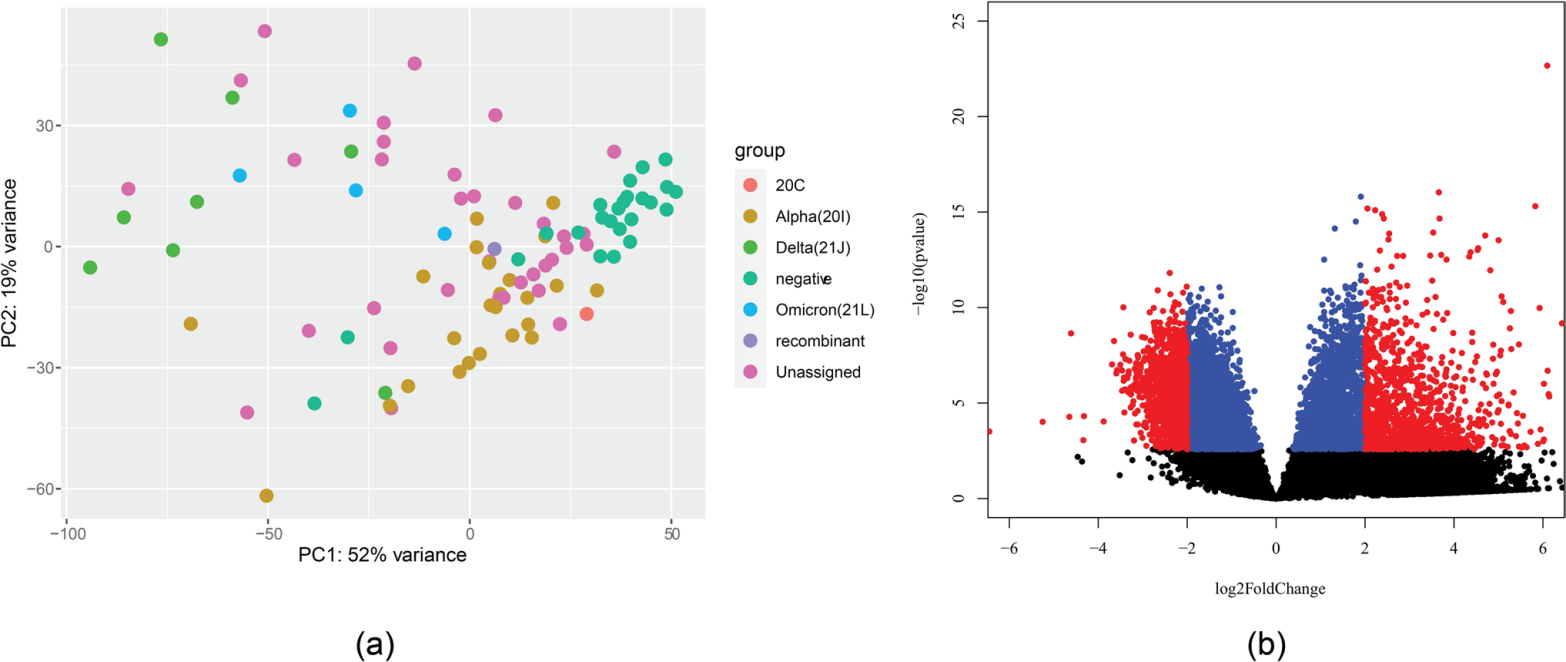
**a** Principal component analysis (PCA) analysis plot of gene expression in different SARS-CoV-2 strains. **b** Volcano plot visualizing differential expression between samples from patients with the Alpha variant (20I) and those with the Delta variant (21 J). Colors show if gene passes through threshold requirements—blue (adj. *p*-value < 0.01) and red (adj. *p*-value < 0.01 and log2 fold change > 2)

Our data also show variation by a specific SARS-CoV-2 variant (based on the WHO variant names). The distance in the transcriptional profile is visualized in Fig. 2a. A large difference was shown when comparing the Alpha (all 20I) and Delta (all 21 J) variants. There were 14,109 genes with *p*-value < 0.1, which is visualized in Fig. 2b. We speculate further on the reasons for this difference in the “Discussion” section. A comparison of patients with Delta and Omicron (albeit with a limited number of samples) brought 468 genes with a *p*-value < 0.1. However, it needs to be pointed out that all Omicron samples were from female patients.

### Pathways altered by the infection

**Fig. 3:**
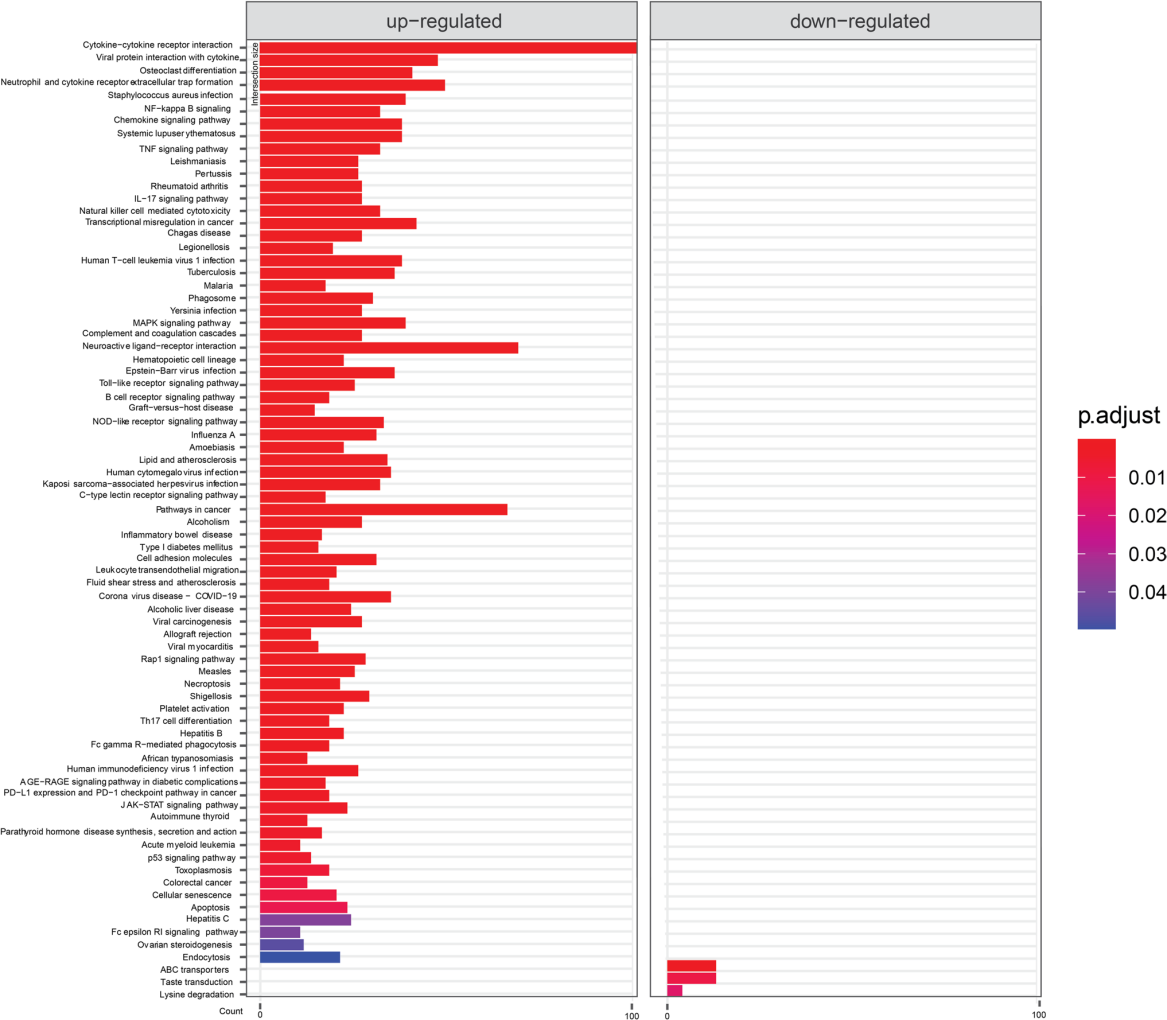
KEGG terms mapping on differentially expressed genes for COVID-19 positive samples. Only the results that meet requirements of the default threshold of adjusted *p*-value < 0.05 are visualized. Length of the bars is showing gene counts for the term it belongs to

Since no unequivocal information can be obtained from the large number of genes that meet the criteria for significant effect, we performed an enrichment analysis of the KEGG pathways. Comparison of COVID-19-positive and COVID-19-negative patients (Fig. 3) showed that most of the pathways comprising enriched genes with up-regulation are related to immune response to various diseases or to other immune-related pathways (cytokine-cytokine receptor interaction, neutrophil extracellular trap formation, viral protein interaction with cytokine, natural killer cell, B cell receptor, NOD-like receptor, Fc gamma R − mediated phagocytosis, Th17 cell differentiation). Other pathways with enriched genes comprised the signaling pathways mostly related to immune system (NF-kappa B signaling pathway, TNF signaling pathway, chemokine signaling pathway, IL-17 signaling pathway, MAPK signaling pathway, C-type lectin receptor signaling pathway, Rap1 signaling pathway, JAK-STAT signaling pathway, p53 signaling pathway). Other KEGG pathways included osteoclast differentiation, cell adhesion molecules, alcoholism, and alcoholic liver disease. For the down-regulated genes, 3 pathways were enriched: ABC transporters, taste transfer, and lysine degradation.

**Fig. 4:**
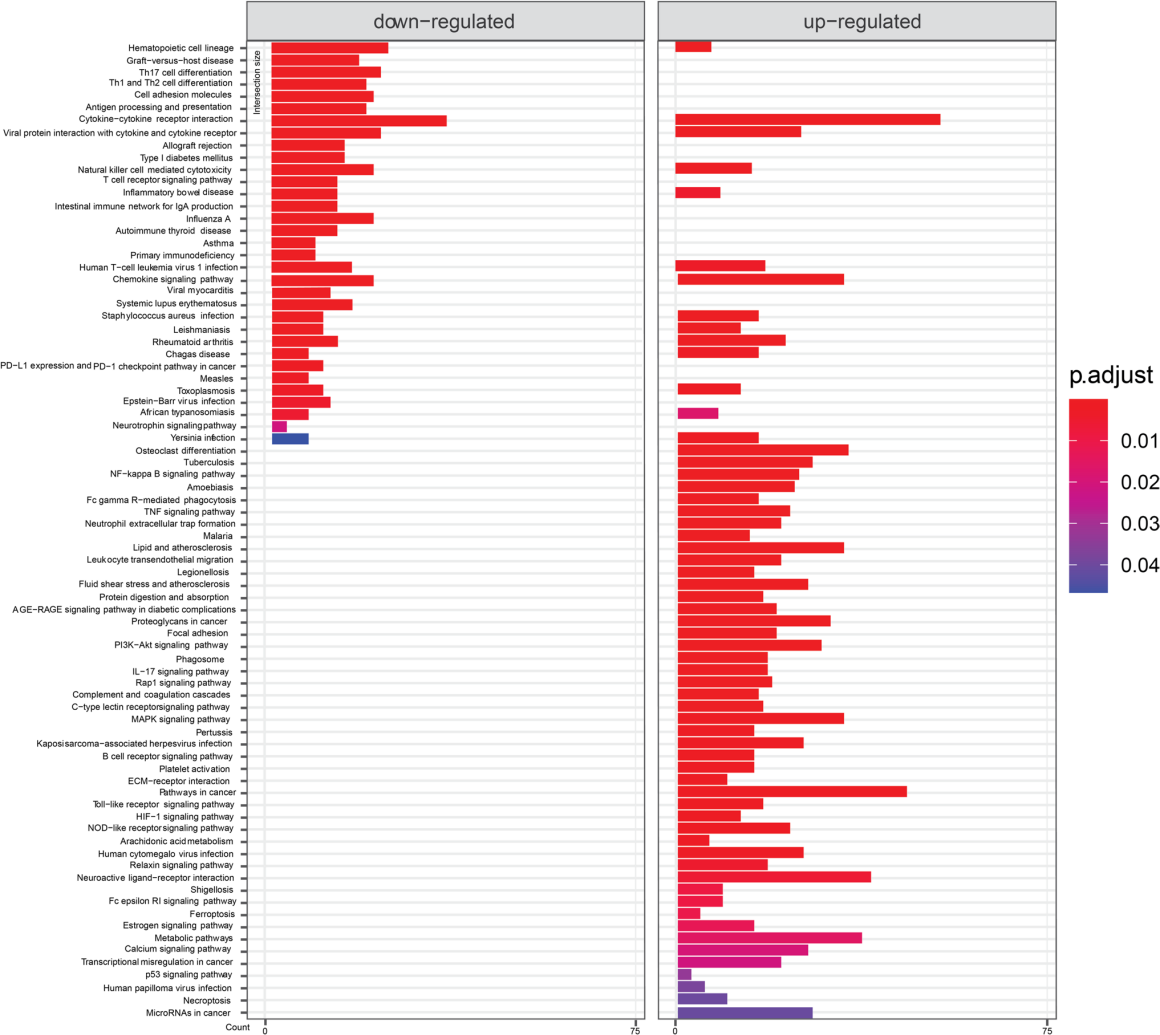
KEGG terms mapping on genes differentially expressed in samples from patients with severe symptoms compared to those with mild symptoms. Only the results that meet requirements of the default threshold of adjusted *p*-value < 0.05 are visualized. Length of the bars is showing gene counts for the term it belongs to

KEGG pathway analysis of mild symptoms compared to severe symptoms is shown in Fig. 4. Again, infectious disease- and immunity-related concepts dominated. Cytokine interactions-related terms were significant for both down- and up-regulated genes. From signaling pathways, there are NF-Kappa B, neutrophin, TNF, PI3K-Akt, IL-17, Rap 1, MAPK, Toll-like receptor, C-type lectin, B cell receptor, HIF-1, NOD-like receptor, calcium, and estrogen-signaling pathways.

## Discussion

This article presents a pilot study of the human nasopharyngeal transcriptome and metatranscriptome of patients with COVID-19. By sequencing samples and subsequent data analysis, we studied differences in gene expression between 72 COVID-19-positive and 24 COVID-19-negative patients, between patients with severe and mild symptoms, and between those who were infected with the Alpha and Delta SARS-CoV-2 variants. We observed substantial changes on transcriptomic levels between the above groups. The most suitable way to present the results appears to be pathway analysis visualization, which demonstrates the involvement of numerous mostly immunity-related pathways, signaling pathways directly or indirectly connected to immunity, and specific disease terms (Figs. 3 and 4). Results point to cytokine, chemokine activity and inflammation. Observation of KEGG terms referring to various diseases (not only COVID-19) might be caused by similarities in the effect on tissue between infections or other disease events.

For COVID-19-linked genes, the most of these terms were pathways with many of the concerned genes up-regulated in samples from COVID-19-positive patients. It seems signaling pathways of the innate immunity were most affected. Genes of three important modulators of innate immunity—Nod-like (NLR), Toll-like receptor (TLR), and C-type lectin receptor (CLR) signaling pathways—were up-regulated. Multiple of other seemingly up-regulated signaling pathways (judging by up-regulation of their genes) are part of processes connected to NLR and TLR signaling. Those are NF-kB signaling, MAPK signaling, and JAK-STAT signaling (Hata et al. 2002; Kawasaki and Kawai 2014). AGE-RAGE signaling genes might be involved thorough activating pathways resulting in NF-kB activity (Tobon-Velasco et al. 2014). Counting mentioned pathways, many up-regulated processes point to elevated cytokine or chemokine activity and promotion of inflammatory processes (Kawasaki and Kawai 2014; Rawlings et al. 2004). Apart from mentioned, it is the case for TNF signaling, chemokine signaling, IL-17 of Fc epsilon RI-mediated signaling (Aggarwal 2003; Hata et al. 2002; Zhu et al. 2004). From remaining significant signaling pathways, p53 signaling is involved in different processes like cell cycle control and apoptosis (Hernández Borrero and El-Deiry 2021). Rap1 signaling have various roles like cell proliferation and adhesion; however, it might contribute through regulating MAPK (Zhang et al. 2017; York et al. 1998). Downregulated pathways identified were only ABC transporters, taste transduction, and lysine degradation.

Severe patients differed from those with mild symptoms with numerous altered pathways as well. In fact, same pathways as those making difference between COVID-19-positive and COVID-19-negative patients seem to be crucial (Fig. 4). However, here some genes of cytokine and chemokine pathways were downregulated in severe patients. Th1, Th2, and Th17 cells differentiation genes were downregulated in severe patients, while Th17 cell differentiation actually seemed supported by up-regulated genes in COVID-19 patients compared to negative donors. PI3K-Akt signaling path-way genes were up-regulated. Other unique results compared to COVID-19 patients in general were estrogen and calcium signaling pathways. It seems severity of COVID-19 is followed by even more inflammatory processes.

There are multiple processes that have been reported in previous studies to be involved in changes caused by infections in general and are regarded as key pathways altered during the SARS-CoV-2 infection. We identified most of those key pathways in pathway enrichment using KEGG, Reactome, and Wiki Pathway databases. KEGG pathway enrichment detected terms related to TLR receptors. In fact, TLR receptors are known to be the key component in reacting to the SARS-CoV-2 infection. For example, TLR3 hyperactivation can lead to a cytokine storm and the subsequent severe COVID-19 (Jamison et al. 2022). Other components, such as interleukins (IL-2, IL-6, IL-7, IL-10) and TNF-α are connected to severe COVID-19 symptoms, where cytokine storm (a sudden increase in cytokines) occurs instead of a healthy immune response (Fricke-Galindo and Falfán-Valencia 2021). TNF signaling pathway was detected as a significant KEGG pathway in both comparisons of disease positivity and symptom severity. Using the REAC pathway analysis of differentially expressed genes in COVID-19-positive cases, we detected interleukin IL-2, IL-7, and IL-10 as well as IL-1, IL-4, IL-13, and IL-36. KEGG pathway enrichment detected IL-17 signaling. Next, NF-kB related terms were enriched for both gene sets (genes affected by COVID-19 severity or positivity) not only using the KEGG database, but also Wiki Pathway and Reactome. It has been reported that severe COVID-19 is characterized by an inflammatory profile dominated by NF-κB activity (Huang et al. 2020). Next, terms related with TGF-β and JAK-STAT signaling were enriched. JAK-STAT signaling was detected from differentially expressed genes in COVID-19-positive patients. TGF-beta terms were recognized by Wiki Pathway enrichment in both positivity and severity-affected gene sets. In fact, the cytokine TGF-β functions as an activator or suppressor of the Janus kinases (JAKs), and these processes are believed to be affected by the studied viral infection (Tan et al. 2020). Another pathway reported to be altered by SARS-CoV-2 infection, the PI3K-Akt signaling pathway (Al-Qahtani et al. 2022; Li et al. 2021), was also detected in our study (for both severity and positivity-affected gene sets).

A recent study of a similar transcriptome analysis of COVID-19-positive patients, comparing positive patients and negative controls using gene enrichment analysis (albeit with a different software: edgeR and Metascape), has been published by Rhoades et al. (2021). The authors mapped differentially expressed genes to GO terms (while we worked with the KEGG pathways, shown in Fig. 3). However, they too reported terms associated with innate and adaptive host defense pathways, which correlates with our results and is not surprising. Similarly, they found terms related to hematopoiesis (with the CD53 and IKZF1 genes reported, which were included in our results too), inflammatory response, B-cell activation, and leukocyte chemotaxis/mobility (with the CCL2 and CCR1 genes reported). They reported interferon-stimulated genes FITM3, and ISG15, which were also identified in our set of potentially affected genes. Other reported genes included type I interferon signaling genes IRF7 and STAT1 (our results failed to detect STAT1), UN-ATP-1 signaling (with reported genes BCL3 and MYD88, also featured in our results). We did not observe neuronal death pathways, nor the SNCA gene mentioned in the paper by Rhoades et al. (however, MDK, another listed gene, can be also found in our results). We did not observe many downregulated genes-related pathways (in the infected samples), whereas Rhoades et al. reported GO terms associated with tissue homeostasis and cellular organization. In the study con-ducted by Bass et al., up-regulation of innate immune response through several inflammatory genes and NF-kB was reported. This corroborates our observations, although samples collected from other organs and not from nasopharyngeal swabs were combined. IL-17 signaling was found to be increased in moderate and severe patients. We found it to be altered in patients with more severe symptoms and COVID-19 in general (Bass et al. 2021). Similar studies were conducted on blood samples (Daamen et al. 2022; Jackson et al. 2022; Thompson et al. 2023). Another study focused also on the comparison between different infections, but the authors also report results on nasopharyngeal swabs transcriptomics in COVID-19. Their pathway analysis showed prominent activation of genes related to interferon (IFN) signaling and IFN-stimulating genes (ISGs), but inhibition of interleukin-6 (IL-6) and IL-8 signaling genes (including IRAK1 and MAP2K7) (Ng et al. 2021).

A comparison of the results between the Alpha, Delta, and Omicron groups allows for certain degree of speculation. We observed significant differences between the Alpha and Delta groups, but we cannot say what exactly caused the difference in gene expression. In terms of patient background, we know that a larger number of Alpha patients experienced mild symptoms, but there were also patients with severe symptoms. Delta patients, on the other hand, experienced severe symptoms or no symptoms at all. There were 15 male and 8 female Alpha patients in our sample. Both younger and older patients were represented in each group. Some of the patients with severe symptoms were treated with antibiotics. Comparison of Delta and Omicron patients (albeit with a limited sample size) resulted in 468 genes with *p*-values < 0.1. KDM5D and DDX3Y had the highest scores, but these genes are not known to be related to immune response processes. However, they are related to spermatogenesis (Akimoto et al. 2008; Navarro-Costa et al. 2010). All Omicron samples happened to be (randomly) from women, so it is impossible to conclude what portion of the results were influenced by this factor in particular. We also did not elucidate the influence of the other significantly and highly expressed genes, CRYBG3 and PHACTR4. However, the moderately expressed CXCL8 is known to be elevated in viral infections, producing protein known as interleukin-8 (Baggiolini and Clark-Lewis 1992).

The study has several potential drawbacks. Patients with severe symptoms had a higher median age (68 years) compared to patients with mild symptoms (37 years), asymptomatic donors (42 years), or COVID-19-negative individuals from the control group (37 years). This is because the majority of patients hospitalized with severe symptoms were elderly, and therefore, the samples were the most accessible in this regard. Overall, 54.3% of the samples were from female patients. Only in the group of severely symptomatic patients, there was some imbalance (69.5% of samples from male patients). Our study has some typical disadvantages of standard bulk sequencing procedure. We did not separate cell types from each other, so signal from RNA-seq is mix from various cells. Also, there are limitations associated with the sampling procedure (such as different personal). For the purpose of minimalization of various confounding effects, multiple procedures at the level of data analysis were performed (quality control and preprocessing of data, data normalization), Also number of samples should be sufficient to answer significant part of biological variability unassociated with studied topic. There are multiple similar studies, which means that many of our results provide value as a validation study. However, many of these studies present results from different types of tissue or from a blood. The samples in our study originated from nasopharyngeal tissue, and there are not so many studies available with this particular characteristic. As a sample, nasopharyngeal tissue has some drawbacks; however, it allows direct analysis of initial infection site instead of late infection sites in the lower respiratory tract.

## Conclusions

In this study, we investigated nasopharyngeal tissue transcriptome changes in COVID-19 disease. We identified pathways altered by the infection as such or its severity. Most of them are probably components of immune response or alterations caused directly by the virus. Many enriched pathway terms related to different diseases suggest similarity between these different pathological events. Then, there were cytokine-related terms, immune cell-related terms, and signaling pathways—most of them corresponding with the results of previous publications covering the studied topic.

### Supplementary Information

Below is the link to the electronic supplementary material.

Supplementary file1 (DOCX 12 KB)

## Data Availability

Data are available on database ENA (European Nucleotide Archive), under project PRJEB62682. https://www.ebi.ac.uk/ena/browser/view/PRJEB62682.
