# Supplementary material for: Analysis of transcriptomics data from COVID-19 patients: a pilot research
Source: Folia Microbiol (Praha). 2024 Jan 19;69(1):155–64. doi: 10.1007/s12223-024-01130-x (PMC10876742; doi:10.1007/s12223-024-01130-x)
Supplement: Supplementary file 1 — Supplementary file1 (DOCX 12 KB) [file 12223_2024_1130_MOESM1_ESM.docx]

**Supplementary table 1:** **Genes affected by COVID-19 infection.** Differentially expressed genes analysis by DESeq2 statistical test – whole result table. Comparison of gene expressions between 72 samples from COVID-19 positive patients and 24 samples from healthy donors. Significance was judged by adjusted p-vlaue, with threshold set on 0.1.

**Supplementary table 2:** **Genes affected by severity of COVID-19.** Differentially expressed genes analysis by DESeq2 statistical test – whole result table. Comparison of gene expressions between 23 samples from COVID-19 positive patients with severe symptoms and 25 with mild symptoms. Significance was judged by adjusted p-vlaue, with threshold set on 0.1.
